# Clinical Value of Various Histological Factors in Cutaneous and Subcutaneous Mast Cell Tumors in 197 Dogs

**DOI:** 10.1111/jvim.70244

**Published:** 2025-10-15

**Authors:** Katherine Boyd, Melanie Dobromylskyj, Imogen Schofield, Dan O'Neill, Celia Figueroa, Owen Davies

**Affiliations:** ^1^ Bristol Vet Specialists Bristol UK; ^2^ Finn Pathologists Norfolk UK; ^3^ CVS Norfolk UK; ^4^ Royal Veterinary College London UK; ^5^ The Ralph Veterinary Referral Centre Marlow UK

**Keywords:** AgNOR, canine, *c‐kit*, Ki67, KIT staining, margins, MCT, mitotic count, recurrence, tumor

## Abstract

**Background:**

Many histological tests have been correlated with outcome in mast cell tumors (MCTs)in dogs, but their statistical independence is uncertain.

**Objective:**

To investigate the clinical value of histological factors in the prognostication of dogs with MCTs.

**Animals:**

One hundred and ninety‐seven dogs with 199 histologically diagnosed cutaneous (*n* = 153) and subcutaneous (*n* = 43) MCTs treated surgically in primary care practice. All had a commercial prognostic panel performed (Patnaik and Kiupel grade, mitotic count, Ki67, AgNOR, KiAg, *c‐kit* mutation in exons 8 and 11 and KIT localization).

**Methods:**

Retrospective cohort study identifying dogs from searching a commercial laboratory's records (January 2017–August 2020). Follow‐up was collected from clinical records. Outcome measures included MCT specific survival (MSS) and recurrence.

**Results:**

Multivariable Cox proportional hazard regression identified only mitotic count > 5 (HR 10.2; 95% CI 3.2–32.8; *p* < 0.001) predicted poorer MSS across all MCTs. In Patnaik grade I or II and Kiupel low‐grade cutaneous MCTs, only *c‐kit* mutation in exon 11 (HR 20.8; 95% CI 1.80–224.8; *p* = 0.015) predicted MSS. A *c‐kit* mutation in exon 11 (HR 10.0; 95% CI 3.0–32.9; *p <* 0.001), age, and histological tumor free margins < 2 mm independently predicted cutaneous and subcutaneous MCT recurrence. In Patnaik grade I or II, and Kiupel low‐grade cutaneous MCTs, *c‐kit* mutation in exon 11 (HR 23.20; 95% CI 2.3–231.3; *p* = 0.007) and AgNOR (HR 13.73; 95% CI 1.6–115.6; *p* = 0.016) predicted MCT recurrence.

**Conclusion and Clinical Importance:**

This study suggests a comparatively greater role of *c‐kit* mutations in exon 11 and AgNOR in the prognostication of MCTs, while Ki67 appears less important.

**Abbreviations**
AgNOR: argyrophylic nucleolar organizer regions
AUC: area under the curve
CI: confidence interval
HR: hazard ratio
HTFM: histological tumor free margin
IQR: interquartile range
MCT: mast cell tumor
MSPS: mast cell tumor specific proportional survival
MSS: mast cell tumor specific survival
MST: median survival time
OS: overall survival
ROC: receiver operating characteristics
SD: standard deviation

## 1 Introduction

Mast cell tumors (MCTs) represent 16% to 20% of all cutaneous and subcutaneous tumors in dogs [1, 2]. Histological grading systems are well‐established in cutaneous MCTs with the Patnaik and Kiupel grading systems in common use. However, over half of cutaneous MCTs are Patnaik grade II and Kiupel low‐grade [3, 4, 5, 6, 7], and these categories remain heterogeneous in clinical outcome; 16.5% of Patnaik grade II and 14.9% of Kiupel low‐grade tumors will metastasize, and 12% to 23% of Patnaik grade II MCTs and 4.7% of Kiupel low‐grade tumors result in death [3, 4, 7, 8]. Grading systems are not routinely used for subcutaneous MCTs, but distinguishing cutaneous and subcutaneous MCTs might not be straightforward. A recent study [9] demonstrated Kiupel grade, along with other histological factors, to be prognostic in both cutaneous and subcutaneous MCTs [9] in contrast to many historic studies [3, 4, 6, 7, 8, 10, 11, 12, 13, 14, 15].

With the limitations of histological grading, many studies have investigated other prognostic factors. Higher mitotic counts (> 5 per 10 high powered fields) are reported as independent of histological grade in predicting MCT‐related death and metastasis [16, 17, 18, 19, 20, 21]. Higher Ki67 indices are reported as independent of histological grade and mitotic count and associated with greater risk of recurrence, metastasis, and poorer survival [22, 23, 24]. In subcutaneous MCTs, higher Ki67 indices are associated with a higher risk of metastasis and recurrence [20]. Increased argyrophylic nucleolar organizer regions (AgNORs)/cell are associated with higher‐grade tumors, greater recurrence, and death [20, 23, 24, 25, 26]. Increased KiAg (product of Ki67 and AgNOR) values are associated with a greater risk of metastasis and death [20, 23]. *c‐kit* mutations in exon 11 are associated with higher grade MCTs and tumors with increased proliferation indices, but have not been shown to be independent [23, 27, 28, 29, 30], however, the *c‐kit* mutation in exon 8 has not been associated with a poorer outcome [28]. Finally, KIT staining patterns II and III are associated with a higher rate of tumor recurrence and are reported in combination with increases in other markers of proliferation [20, 23, 29].

Many previous studies have investigated single markers in relation to outcome or histological grade. Very few studies have utilized multivariable statistical analysis on a panel of prognostic tests. The aim of this study was to investigate the prognostic value of the aforementioned histological and immunohistochemical prognostic factors (in predicting MCT‐specific survival and time to MCT recurrence) in a group of cutaneous and subcutaneous MCTs in dogs, analyzed separately and together, and to identify prognostic factors in Patnaik grade II, Kiupel low grade MCTs. Secondary aims were to investigate correlations between prognostic factors, describe the outcome of dogs with discordant prognostic factor results, and investigate histological tumor free margins (HTFM) in relation to MCT recurrence. Our hypothesis was that cutaneous tumor grade (Patnaik and Kiupel), mitotic count > 5 (in 2.37 mm^2^) and Ki67 index > 23 (per grid area) would be significant in a multivariable statistical model while *c‐kit* mutations, KIT patterns, and AgNOR scores would have a significant, but non‐independent effect.

## 2 Materials and Methods

This was a retrospective cohort study. A search of the clinical submission database of a large UK‐based commercial diagnostic laboratory (Finn Pathologists, https://www.finnpathologists.com/) was undertaken to identify dogs that had undergone surgical excision (at their primary care practice) for a mass which histopathology documented to be a cutaneous or subcutaneous MCT and had then had a commercially available panel of prognostic markers performed, between January 2017 and August 2020. This prognostic panel included histological grade (Patnaik and Kiupel grading systems), mitotic count (number of mitotic figures in 2.37 mm^2^), Ki67 (average per grid area [23]), AgNOR (average per cell), KiAg, KIT staining pattern, and *c‐kit* mutation on exons 8 and 11. Dogs with MCTs at the mucocutaneous junction or visceral MCTs were excluded. Histopathology was not reviewed for the purposes of this study.

Clinical follow‐up data were collected (between August and September 2022) through direct access to each dog's primary care practice record via the use of the VetCompass database; this database allows direct access to the animal clinical records (but not sensitive client information) of linked primary care practices for research purposes [31]. A total of 206 cases were identified based on the search of the laboratories records; VetCompass records were entirely blank in 6 cases, and one individual ID linked to a horse's clinical notes. These 7 cases were excluded, leaving 199 for inclusion. Where available, the following data were collected: dog age, breed, sex, the date when the tumor was first noted, date of surgery, anatomical location, cutaneous or subcutaneous location, any concurrent medications (including pre‐operative glucocorticoids) and comorbidities, abdominal and thoracic imaging undertaken, cytology of lymph nodes, liver, and spleen (where performed), adjunctive medical treatment, MCT recurrence (and date), along with any subsequent treatment, date of death, and reason (if known) or date of last follow‐up. Where dogs were alive, lost to follow‐up, or cause of death was unclear, unrecorded, or not due to MCT, they were censored on the date of last known follow‐up. Breed was analyzed separately and grouped according to branches of the canine cladogram [32, 33] due to the large number of represented breeds. Outcomes measured were MCT‐specific survival (MSS), overall survival (OS) and time to tumor recurrence or progression. Cause of death was recorded either as MCT‐related, where clinical records showed this was clearly due to local or metastatic disease, or not MCT related, if the cause of death was unclear or unrelated to the MCT. Survival times were calculated from the date the excised tumor was submitted to the laboratory. Dates of recurrence, progression, and death were extracted as they were reported in the clinical notes and used to calculate median survival time (MST) and MCT‐specific proportional survival (MSPS). Histological margins were analyzed to assess the risk of recurrence and categorized in two different ways. First, as incomplete (tumor cells on the surgical margin) and complete (all histological margins > 0 mm) [34, 35, 36, 37] and second, as three categories: incomplete, > 0 mm to < 2 mm, and ≥ 2 mm [15, 38, 39], based on previous publications of soft tissue sarcomas in dogs.

An a priori sample size calculation using Stata 17 (Stata, TX, USA) estimated that for a Cox proportional hazards analysis, a minimum of 178 dogs would be required to observe a hazards ratio of at least 3.5 at 5% significance, 80% power, with an expected MCT attributable mortality proportion of 15% at the end of the study period and a 25% loss to follow up. The hazard ratio of 3.5 was based on the aim to detect clinically useful information; in clinical trials, a hazard ratio of 3.5 or above is considered clinically significant, and previous MCT publications have reported hazard ratios greater than this [21, 40, 41, 42]. The 15% estimate for MCT‐related death was based on analysis of pilot data and previous studies suggesting a 2‐to‐5 year survival in 85%–87% of dogs with MCTs [11, 21].

## 3 Statistical Analysis

Stata 17 (Stata, TX, USA) was used for statistical analysis. Quantitative data were assessed for normality using the Shapiro–Wilk test and graphical assessment. Parametric data were summarized using the mean and standard deviation (SD) and non‐parametric data using the median, interquartile range (IQR) and range.

The time‐to‐event outcome data included MSS, OS, and MCT recurrence. Survival (or time‐to‐event) distributions were summarized by the median survival time and cumulative survival proportion. Kaplan–Meier plots explored the time‐to‐event outcomes. Data for cutaneous and subcutaneous MCTs were analyzed separately and as a single group based on a recent publication [9].

Univariable and multivariable Cox proportional hazard modeling assessed associations between cohort demographics (such as age, breed, sex), recorded risk factors (such as location, histological grade, mitotic count, Ki67, AgNOR, *c‐kit* mutation in exon 11 and KIT staining pattern) and the time‐to‐event outcomes. Age was analyzed as a continuous variable in all statistical models. Previously established cut‐offs for the histological prognostic factors (mitotic count, Ki67, AgNOR and KiAg) were utilized in the initial statistical models [17, 18, 20, 23]. KIT staining pattern was determined based on previously established criteria [29]. All potential associations were initially assessed in univariable models. Missing data (due to an incomplete record) was handled by excluding that case when analyzing the specific variable. To assess the prognostic markers that are most predictive of survival when multiple prognostic markers are presented within a pathology report, a multivariable prediction model was developed. Risk factors in the univariable analysis with *p* < 0.20 were carried forward for multivariable modeling, taking multiple risk factors and demographics into account. In the two dogs with two MCTs, the latter dated mass was removed from the regression analysis. Pairwise correlations between prognostic factors were explored to identify potential collinearity using Pearson's correlation coefficient for continuous risk factors. Prognostic factors were considered highly correlated if *r* > 0.80. High correlation between two dichotomous variables was assessed with univariable logistic regression and was considered collinear if this resulted in extreme values of odds ratios (OR > 10). If two prognostic markers were considered collinear, the marker with the strongest association was retained for multivariable assessment. A backwards stepwise manual approach was used to build the multivariable model, with predictive factors kept in the final model after assessing for effects of confounding and if they were associated with the outcome of interest (*p* < 0.05). Two sets of multivariable models were built; including cutaneous tumors only and including both cutaneous and subcutaneous tumors, the latter excluding histological grades. A final model was created including only those tumors that were graded at Patnaik grade II and Kiupel low grade to identify prognostic factors in this group. The proportional hazards assumption was assessed by visual inspection of log‐minus‐log survival plots and by statistical assessment of Schoenfeld residuals.

Agreement between two categorized (based on previously published cut‐offs [17, 18, 20, 23]) prognostic markers was compared with Cohen's Kappa agreement. Cohen's Kappa was interpreted as: ≤ 0 indicated no agreement, 0.01 to 0.20 was slight agreement, 0.21 to 0.40 was fair, 0.41 to 0.60 was moderate, 0.61 to 0.80 was substantial, and 0.81 to 1.00 was almost perfect agreement [43].

Receiver operating characteristic (ROC) analysis was performed to calculate sensitivity and specificity for different ‘cut‐off’ values for the included factors and to identify optimum cut off values for this study sample aiming for an area under the curve (AUC) > 0.8. The cut‐off was selected by minimizing the distance between the selected point and the point representing perfect classification [44].

## 4 Results

One hundred and ninety‐nine tumors from 197 dogs were identified. Crossbreed (*n* = 39) was the most common breed category; of the pure‐bred dogs, the most common breeds were the Labrador Retriever (*n* = 29), Staffordshire Bull Terrier (*n* = 22) and Boxer (*n* = 13). Breed was unspecified in 20 MCTs. The mean age of diagnosis was 7.6 years (SD 7.7; range 0.9–15.7 years). One hundred and fifty‐three (76.9%) tumors were cutaneous, 43 (21.6%) subcutaneous, and 3 (1.5%) were unspecified. The anatomical locations of these unspecified tumors were described as lateral thigh, lateral thorax, and pinna, and therefore considered to be cutaneous or subcutaneous in location. Twenty‐three dogs (11.7%) had one or more regional lymph nodes sampled with metastasis detected in 8 dogs (34.7%). Nineteen dogs (9.6%) underwent fine needle aspiration of their spleen with metastasis detected in 1 dog (5.2%). Twenty‐one dogs (10.6%) had fine needle aspiration of their liver with metastasis detected in 1 dog (4.7%). Thirty‐eight dogs (19.3%) underwent thoracic imaging, and 60 dogs (30%) underwent abdominal imaging. No dogs underwent bone marrow aspiration. The histological grades and prognostic panel results are presented in Table 1. Due to the low number (*n* = 4) of Patnaik grade I tumors, these were grouped with grade II tumors for statistical analysis. None of the tumors were positive for the *c‐kit* mutation in exon 8. Twenty‐six dogs (13.1%) had adjunctive medical therapy; medical therapies utilized included toceranib (*n* = 6), masitinib (*n* = 12) and vinblastine (*n* = 15) with or without prednisolone. One dog had adjunctive radiation therapy. Median follow‐up time was 727 days (IQR 377–1091 days). Median survival time (MST) for the whole group was not reached. Eighteen dogs (9.0%) were considered to have died or been euthanized due to their mast cell tumor. Forty‐four (22.1%) dogs died or were euthanized due to other causes or for unknown reasons. Fifty dogs were lost to follow up, and 87 dogs were alive at the time of data collection. Across all MCTs, one‐year MSPS was 93% (95% confidence interval (CI) 88%–96%) and four‐year MSPS was 86% (95% CI: 76%–92%). Considering the cutaneous cases alone, one‐year MSPS was 92% (95% CI: 86%–96%) and four‐year MSPS was 83% (95% CI: 70%–91%).

**TABLE 1:** Descriptive data from 199 mast cell tumors in 197 dogs.

Variable	Category	Number of MCTs (%)
Tumor location	Cutaneous	153 (76.9)
	Subcutaneous	43 (21.6)
	Unspecified	3 (1.5)
Histological grade (cutaneous only)	Patnaik grade I, Kiupel low grade	4 (2.0)
	Patnaik grade II, Kiupel low grade	120 (60.3)
	Patnaik grade II, Kiupel high grade	14 (7.0)
	Patnaik grade III, Kiupel high grade	13 (6.5)
	Unspecified (subcutaneous tumors, or unspecified location)	46 (23.1)
	Unspecified (data not available)	2 (1.0)
Mitotic count (per 10 high powered fields)	≤ 5	174 (87.4)
	> 5	22 (11.1)
	Unspecified	3 (1.5)
Mitotic count (per 10 high powered fields)	< 2	126 (63.3)
	≥ 2	70 (35.2)
	Unspecified	3 (1.5)
Ki67 (mean per grid area)	≤ 23	136 (68.3)
	> 23	49 (24.6)
	Unspecified	14 (7.0)
AgNOR (mean per cell)	< 2.25	133 (66.8)
	≥ 2.25	62 (31.2)
	Unspecified	4 (2.0)
KiAg	< 54	150 (75.4)
	≥ 54	45 (22.6)
	Unspecified	4 (2.0)
KIT pattern	Pattern 1 (membranous)	49 (24.6)
	Pattern 2 (stippled)	139 (69.8)
	Pattern 3 (diffuse)	7 (3.5)
	Unspecified	4 (2.0)
*c‐kit* mutation on exon 11	Positive	18 (9.0)
	Negative	176 (88.4)
	Unspecified	5 (2.5)

### 4.1 Survival Analysis

Univariable Cox proportional hazards regression performed on the clinical data for MSS is presented in Table 2. This model was also repeated for overall survival, but there was no change in the factors achieving significance (*p* < 0.05). Age and use of adjunctive medical therapy were significantly associated with MSS and overall survival in both cutaneous and subcutaneous tumors when analyzed separately and together.

**TABLE 2:** Univariable Cox proportional hazards regression considering clinical and demographic parameters with relation to mast cell tumor specific survival (including cutaneous and subcutaneous MCTs).

Variable	Category	Number of dogs	MCT specific survival Hazard ratio	95% Confidence interval	*p*
Sex	Female	98	—		
	Male	95	1.60	0.62–4.13	0.333
	Unspecified	6			
Age (years)	Continuous (per year)	199	1.39	1.17–1.65	**< 0.001**
	Unspecified	0			
Breed (> 10 dogs)	Labrador retriever	29	—		
	Boxer	13	0.92	0.35–2.38	0.462
	Staffordshire Bull Terrier	22	0.74	0.33–1.65	0.713
	Crossbreed	39	0.41	0.19–0.89	0.267
Breed group	Terrier/brachycephalic	60	—		
	Retriever/mountain dog	42	1.33	0.38–4.60	0.652
	Spaniel/Poodle	23	1.55	0.37–6.50	0.548
	Pastoral including German shepherd dogs, Shar Pei and Husky	13	1.04	0.12–0.96	0.968
	Crossbreed	39	1.21	0.33–4.53	0.774
	Unspecified	22			
Anatomical area	Trunk	74	—		
	Limbs	55	1.11	0.34–3.65	0.859
	Head/neck (not muzzle)	16	1.41	0.28–7.02	0.674
	Other Including digit (*n* = 4); muzzle (4); prepuce (haired skin; 5); vulva (haired skin; 1); perineum (2); scrotum (3) and tail (2)	21	1.01	0.20–5.05	0.987
	Unspecified (haired skin)	33	N/A		
Location	Cutaneous	153	—		
	Subcutaneous	43	0.47	0.12–2.04	0.310
	Unspecified	3			
Adjunctive medical therapy	Yes	26	—		
	No	173	0.15	0.06–0.38	**< 0.001**

*Note:* Breeds are included where > 10 dogs are represented, a full list of breeds can be found in supplementary table 1. *p* Values highlighted in bold when < 0.05.

**FIGURE 1:**
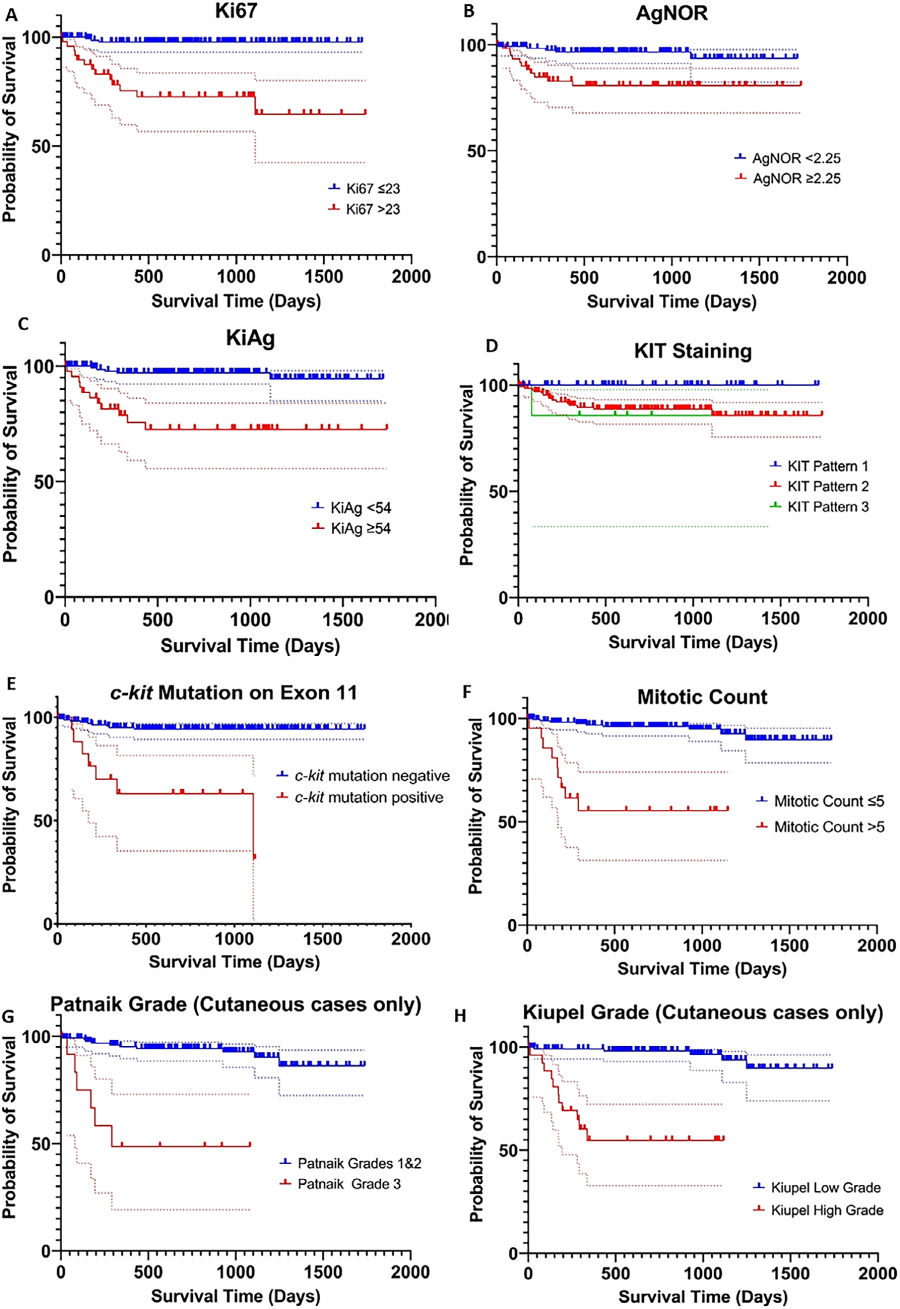
Kaplan Meier survival curves of mast cell specific survival from the date of laboratory submission for each prognostic factor (*n* = 199 tumors). 95% confidence intervals included in the dotted lines. Curves g and h include cutaneous cases only because histological grades are only validated in cutaneous tumors (*n* = 153).

Cox proportional hazards regression analysis of prognostic panel data for MSS forced in age and use of adjunctive therapy as a priori confounding factors. This allowed the calculation of adjusted (for age and use of adjunctive medical therapy) hazard ratios for each of the prognostic tests with respect to MSS (Table 3). All of the prognostic factors used in the prognostic panel were associated with greater hazard ratios for MCT‐related death except for KIT staining pattern. The Kaplan–Meier survival plots for the prognostic data are presented in Figure 1.

**TABLE 3:** Cox proportional hazards regression model for individual prognostic tests for mast cell tumor specific survival.

		Cutaneous and subcutaneous MCT cases *n* = 199				Cutaneous MCT cases *n* = 153			
Variable	Category	Number of MCTs	MCT attributable mortality Hazard ratio	95% Confidence interval	*p*	Number of MCTs	MCT attributable mortality Hazard ratio	95% Confidence interval	*p*
Patnaik grade	1 or 2	138	—			138	—		
	3	13	15.42	4.50–52.83	**< 0.001**	13	15.42	4.50–52.83	**< 0.001**
Kiupel grade	Low	124	—			124	—		
	High	27	22.79	5.51–94.29	**< 0.001**	27	22.79	5.51–94.29	**< 0.001**
Mitotic count	≤ 5	174	—			131	—		
	> 5	22	15.43	5.35–44.50	**< 0.001**	21	15.58	4.59–52.90	**< 0.001**
Mitotic count	< 2	126	—			95	—		
	≥ 2	70	8.49	2.41–30.0	**0.001**	57	5.30	1.45–19.42	**0.012**
Ki67	≤ 23	136	—			111	—		
	> 23	49	7.60	2.38–24.27	**0.001**	40	12.55	2.67–58.74	**0.001**
AgNOR	< 2.25	133	—			99	—		
	≥ 2.25	62	6.74	2.14–21.23	**0.001**	52	6.43	1.77–23.33	**0.005**
KiAg	< 54	150	—			115	—		
	≥ 54	45	8.68	2.89–26.06	**< 0.001**	36	12.83	3.32–49.66	**< 0.001**
KIT staining pattern	1	49	0.13	0.02–1.06	0.057	30	Too few		
	2	139	—			115	Too few		
	3	7	1.51	0.20–11.59	0.692	6	Too few		
*c‐kit* mutation on exon 11	Positive	18	7.69	2.78–21.28	**< 0.001**	17	6.85	2.20–21.25	**0.001**
	Negative	176	—			136	—		

*Note:* Hazard ratios are adjusted for age and use of adjunctive medical therapy. Cutaneous and subcutaneous cases are grouped (left; *n* = 199 tumors), and cutaneous cases are presented alone (right; *n* = 153 tumors). *p* Values highlighted in bold when < 0.05.

Multivariable models were built for MSS incorporating both the clinical and histological data of all MCTs; cutaneous MCTs were also analyzed as a separate group. In the model considering both cutaneous and subcutaneous tumors (therefore not including histological grade), mitotic count > 5 (HR 10.2; 95% CI 3.2–32.8; *p* < 0.001) and increasing age (per year) (HR 1.5; 95% CI 1.2–1.9; *p* < 0.001) were associated with poorer MSS. Not utilizing adjunctive chemotherapy (HR 0.34; 95% CI 0.1–0.9; *p* = 0.049) was associated with superior MSS. In cutaneous tumors, histological grade, age, and use of adjunctive chemotherapy were significant in the final model. Of the prognostic markers, only *c‐kit* mutation in exon 11 came close to achieving statistical significance (*p* = 0.051) and was retained in the final model, Table 4. Considering only those tumors graded as Patnaik grade I or II and Kiupel low grade, only the presence of *c‐kit* mutation in exon 11 retained significance in the multivariable model for predicting MSS (HR 20.8; 95% CI 1.80–224.8; *p* = 0.015).

**TABLE 4:** Multivariable Cox proportional hazards regression model for cutaneous MCT specific survival (*n* = 148).

Prognostic factor	Category	Number of MCTs	MCT attributable mortality Hazard ratio	95% Confidence interval	*p*
Patnaik grade	1 or 2	135	—		
	3	13	8.33	2.42–28.66	**0.001**
*c‐kit* mutation on exon 11	Positive	17	3.18	1.00–10.14	0.051
	Negative	131	—		
Use of adjunctive chemotherapy	Yes	21	—		
	No	127	0.21	0.06–0.71	**0.012**
Age	Continuous (per year)	148	1.54	1.16–2.05	**0.003**

*Note:* *p* Values highlighted in bold when < 0.05.

### 4.2 ROC Curve Analysis

ROC curve analysis was used to determine optimum cut‐off points for the prognostic data in this study cohort for predicting MCTs resulting in the death of the dog (cutaneous and subcutaneous combined). A mitotic count of ≥ 2 in 10 high‐powered fields had 63% sensitivity and 67% specificity (AUC 0.79) for predicting tumor‐related death, compared to 53% sensitivity and 90% specificity using a mitotic count of > 5. A Ki67 ≥ 20 positive cells per grid area gave 76% sensitivity and 79% specificity (AUC 0.8). AgNOR ≥ 1.97 per cell had 87% sensitivity and 52% specificity (AUC 0.75). KiAg ≥ 40.95 had 78% sensitivity and 79% specificity (AUC 0.81). The multivariable statistical models were re‐run with the cut‐offs calculated by ROC curve analysis, but there was no change in the factors retaining significance.

### 4.3 Agreement Between Prognostic Factors

Table 5 shows the results of the Cohen's Kappa analysis comparing the agreement between the prognostic factors. All comparisons had some level of agreement. Only slight to fair agreement was seen between *c‐kit* mutation status in exon 11 and AgNOR (Kappa (*K*) = 0.2), Ki67 (*K* = 0.23) and KiAg (*K* = 0.22). There was substantial agreement between mitotic count (using ≤ 5 and > 5 as the cut‐offs) and both histological grading systems. However, when a cut‐off for mitotic count of 2 was used, there was moderate agreement with the Kiupel grading system and fair agreement with the Patnaik system.

**TABLE 5:** Cohen's Kappa analysis comparing the agreement between pairs of prognostic factors. This was interpreted as: ≤ 0 was no agreement, 0.01–0.20 was slight agreement (green), 0.21–0.40 was fair (yellow), 0.41–0.60 was moderate (orange), 0.61–0.80 was substantial (light red), and 0.81–1.00 was almost perfect agreement (red). MC2 – mitotic count cut off ≥ 2, MC5 – mitotic count cut off > 5.

	Kiupel high grade	MC (> 5 cut off)	Ki67 > 23	AgNOR ≥ 2.25	KiAg≥ 54	*c‐kit* mutation in exon 11	Patnaik grade 3
Kiupel high grade							
MC (> 5 cut off)	0.75						
Ki67 > 23	0.59	0.36					
AgNOR ≥ 2.25	0.35	0.35	0.54				
KiAg ≥ 54	0.57	0.39	0.94	0.58			
*c‐kit* mutation in exon 11	0.49	0.50	0.23	0.20	0.22		
Patnaik Grade 3	0.58	0.72	0.31	0.30	0.35	0.33	
MC (≥ 2 cut off)	0.51	0.38	0.49	0.29	0.43	0.24	0.28

**FIGURE 2:**
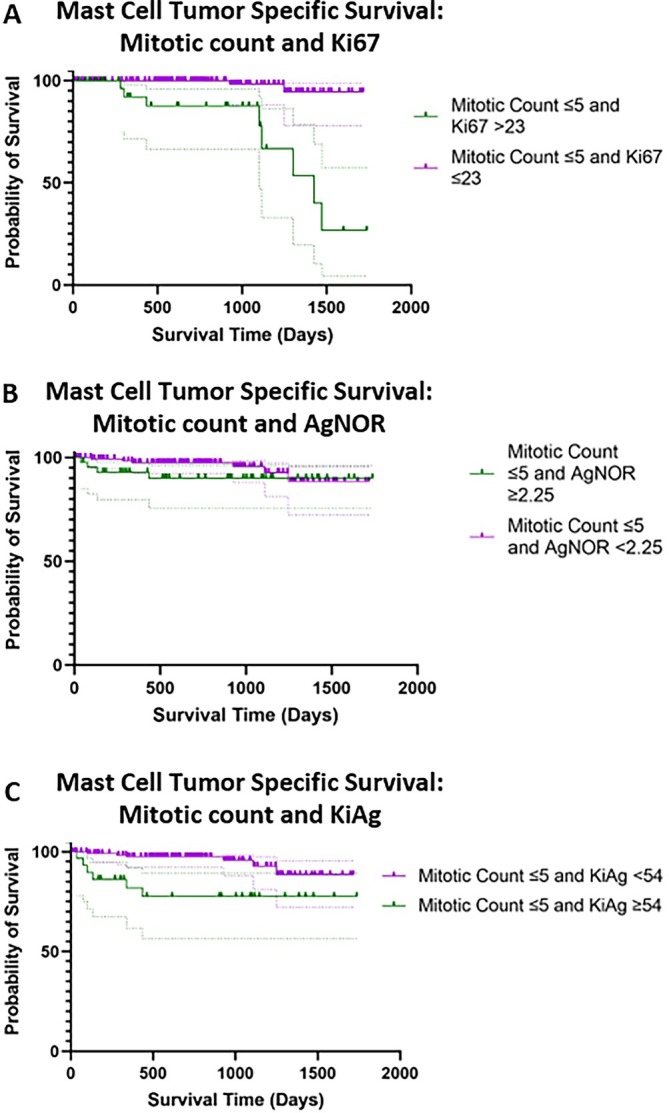
Kaplan Meier survival curves for discordant proliferation indices across both cutaneous and subcutaneous tumors; 95% confidence intervals are depicted by the dotted lines.

Thirty‐three tumors (from 33 dogs) had a mitotic count ≤ 5 and a Ki67 > 23; of these dogs, 6 (18%) died due to their MCT within 1 year of surgery. Twelve dogs (36%) had a mitotic count < 2 and a Ki67 > 23; only one dog experienced progression with metastasis to the liver and spleen being identified 11 months after surgery; the dog was subsequently euthanized. Six dogs (18%) had a mitotic count > 5 and a Ki67 ≤ 23; 4 of these dogs died due to their MCT, all within 1 year of surgery. The other two dogs were euthanized; one with a splenic mass (not sampled) 350 days post‐surgery and the other from unknown reasons (> 1000 days following surgery). Forty‐three tumors had a mitotic count ≤ 5 and AgNOR ≥ 2.25/cell; 3 (7%) of these dogs died within 1 year of surgery, and the MST was not reached. Kaplan Meier plots, including 95% confidence intervals, for the larger discordant groups are presented in Figure 2.

### 4.4 Recurrence Analysis

Twenty tumors recurred after surgery. All the prognostic factors (Patnaik grade III, Kiupel high grade, mitotic count > 5 and ≥ 2, Ki67 index > 23, AgNOR ≥ 2.25, KiAg ≥ 54, *c‐kit* mutation in exon 11, KIT staining pattern 2 or 3), incomplete histological margins, and HTFM < 2 mm were significantly associated with a greater risk of recurrence on univariable analysis. There was no statistically significant difference in recurrence between cases with incomplete excision and HTFM < 2 mm (Table 6). Sex, age, breed/breed group, anatomical area, and cutaneous vs. subcutaneous tumors were not associated with the risk of recurrence. In the multivariable model considering all tumors together and removing histological grades from the analysis, *c‐kit* mutation status in exon 11 and age were associated with a greater risk of recurrence, whereas HTFM ≥ 2 mm was associated with a lower risk of recurrence (Table 7).

**TABLE 6:** Univariable Cox proportional hazards regression model for MCT recurrence (including cutaneous and subcutaneous MCTs).

Variable	Category	Number of tumors	MCT recurrence Hazard ratio	95% Confidence interval	*p*
Adjunctive medical therapy	Yes	26	—		
	No	173	0.19	0.08–0.48	**< 0.001**
Margins	Incomplete	51	0.33	0.12–0.91	**0.033**
	Complete (> 0 mm)	148	—		
Margins	Incomplete	51	—		
	> 0 – < 2 mm	42	0.68	0.20–2.26	0.527
	≥ 2 mm	88	0.25	0.08–0.84	**0.024**
	Not measured	18			
Patnaik grade (cutaneous MCTs)	1 or 2	138	—		
	3	13	8.34	2.54–27.37	**< 0.001**
Kiupel grade (Cutaneous MCTs)	Low	126	—		
	High	27	14.57	4.92–43.15	**< 0.001**
Mitotic count	≤ 5	174	—		
	> 5	22	12.24	4.82–31.06	**< 0.001**
Mitotic count	< 2	126	—		
	≥ 2	70	8.17	2.72–24.49	**< 0.001**
Ki67	≤ 23	136	—		
	> 23	49	8.50	3.03–23.88	**< 0.001**
AgNOR	< 2.25	133	—		
	≥ 2.25	62	8.22	2.70–24.98	**< 0.001**
KiAg	< 54	150	—		
	≥ 54	45	5.75	2.22–14.86	**< 0.001**
KIT staining pattern	1	49	0.12	0.01–0.91	**0.040**
	2	139	—		
	3	7	1.14	0.15–8.63	0.896
*c‐kit* mutation on exon 11	Positive	18	10.49	3.86–28.48	**< 0.001**
	Negative	176	—		

*Note:* *p* Values highlighted in bold when < 0.05.

**TABLE 7:** Multivariable Cox proportional hazards regression model for MCT recurrence (including cutaneous and subcutaneous MCTs).

Prognostic factor	Category	Number of tumors	MCT recurrence Hazard ratio	95% Confidence interval	*p*
*c‐kit* mutation on exon 11	Positive	18	10.01	3.04–32.95	**< 0.001**
	Negative	163	—		
Age	Continuous (per year)	181	1.31	1.08–1.58	**0.006**
Margins	Incomplete	51	—		
	> 0 – < 2 mm	42	0.43	0.11–1.63	0.212
	≥ 2 mm	88	0.19	0.05–0.71	**0.014**

*Note:* *p* Values highlighted in bold when < 0.05.

Considering cutaneous tumors only, Kiupel high grade (HR 13.43; 95% CI 2.0–88.7; *p* = 0.007), AgNOR counts ≥ 2.25/cell (HR 7.57; 95% CI 1.18–48.65; *p* = 0.033) and increasing age (per year) (HR 1.46; 95% CI 1.1–1.9; *p* = 0.009) were associated with a greater risk of MCT recurrence. There were too few subcutaneous MCTs to assess as an individual group.

In those tumors graded as Patnaik grade I or II, and Kiupel low grade, the presence of a *c‐kit* mutation in exon 11 (HR 23.20; 95% CI 2.3–231.3; *p* = 0.007) and AgNOR counts ≥ 2.25/cell (HR 13.73; 95% CI 1.6–115.6; *p* = 0.016) were independently significant for MCT recurrence. There was no difference in the risk of recurrence between tumors with incomplete histological margins and those with HTFM < 2 mm. Only tumors with HTFM ≥ 2 mm had a lower risk of recurrence in the multivariable model.

## 5 Discussion

This was a retrospective study aiming to investigate the independence of prognostic factors presented in a commercially‐available prognostic panel for MCTs in dogs. All the histological prognostic factors had some level of agreement, indicating a degree of collinearity. Considering MSS, other than Patnaik grade, only the presence of a *c‐kit* mutation in exon 11 came close to being independently significant in cutaneous MCTs. Considering cutaneous and subcutaneous MCTs as a group, mitotic count was the only histological parameter that retained significance as an independent predictor of MSS. Considering recurrence among cutaneous tumors, AgNOR counts > 2.25/cell were independent of the other histological features (including histological grade). Among all tumors, the *c‐kit* mutation in exon 11 was independent of the other histological features and there was no statistically significant difference in recurrence between dogs with incomplete excision and HTFM < 2 mm.

This study suggests a greater clinical value of *c‐kit* mutation in exon 11 and AgNOR in the prognostication of MCTs in dogs than previous studies, which have suggested the co‐occurrence of these factors with higher grade tumors but not documented independence [20, 21, 23, 27, 28, 29, 30]. This current study has not identified a single factor with consistently high predictive prognostic value in the different groups examined. Remarkably, contrary to other publications [22, 23, 24] Ki67 was not statistically independent in any of the analyses in this study sample. Several previous studies have reported Ki67 to be significantly prognostic and independent of tumor grade [20, 22, 23]. One possible explanation for this is that the panel of markers and histological grading used in the current study is a broader panel than many previous studies. Including a larger panel of markers that have a degree of collinearity will make identifying a single independent variable more challenging. Furthermore, different laboratories might utilize different Ki67 staining protocols and methods, which could lead to a degree of variability. Certainly, considering the poorer MSS of some cases with discordant mitotic counts and Ki67 indices suggests that Ki67 continues to have a role in the prognosis of some MCTs.

Interestingly, the statistical models in the current study looking at cutaneous MCTs alone and cutaneous and subcutaneous MCTs combined yielded very similar results suggesting they might have similar biological behavior and could be treated similarly. This is in agreement with the recent study by Sabattini et al. [9] which also reported that cutaneous and subcutaneous tumors could behave similarly. Previously, studies have suggested that subcutaneous MCTs might be associated with prolonged survival [21, 26] but more recent studies have reported more aggressive biological behavior in some subcutaneous MCTs [42, 45]. However, the number of subcutaneous tumors was small in the current study; therefore, further research is needed.

A mitotic count cut‐off of 2 per 10 high powered fields has previously been reported to have a sensitivity of 75% and specificity of 80% for predicting tumor‐related death [46]. In the present study sample, this lower mitotic count threshold also appeared to be better at discriminating tumors at higher risk of tumor‐related death from lower risk tumors (compared to a cut off of 5 in 10 high powered fields), both the sensitivity and specificity were lower than previously reported. This could reflect a difference between the sample under study; for example, this study used a cohort of dogs from primary care practice, whereas the study by van Lelyveld et al. was a referred cohort [46].

Although not a primary aim for the current study, HTFM were also included in recurrence analysis. The main finding here was that incomplete excision, versus HTFM < 2 mm, did not predict significant differences in recurrence. However, this should be interpreted with caution since the type of tissue making up the margin (e.g., fascia, intact muscle layer or fat) was not recorded in the current study. Previous studies in veterinary oncology have reported inconsistent findings regarding close surgical margins, with many incompletely excised tumors showing no evidence of recurrence with study [47, 48, 49]. Additionally, it is reported that up to 11% of completely excised MCTs can recur [50] highlighting the need for further research.

Greater age was associated with a significantly higher risk of MCT‐related death in the multivariable models. This finding is in line with previous studies that have also reported poorer outcomes for older dogs [12, 42, 51]. The reasons for this are unclear, but it might be that owners are less motivated to pursue more radical surgeries or adjunctive therapies in an older dog who could have significant comorbidities. Older dogs might also be more likely to develop higher‐grade MCTs [12]. Use of adjunctive therapy was also associated with shorter survival times in this group of dogs. This might be explained by reverse causality; the majority of dogs did not have life‐limiting tumors, and only the dogs identified as being at higher risk of death were treated with adjunctive therapy. This phenomenon is reflected across previous retrospective studies [11, 42].

Breed, anatomical location, and cutaneous vs. subcutaneous location were not associated with survival or recurrence in this study. Some previous studies have suggested that these factors could play a role in prognostication, but these findings are not consistent across all MCT studies [10, 12, 21, 52, 53, 54, 55]. This study sample might not have included sufficient numbers of dogs with MCTs in the previously reported higher‐risk locations, as well as the majority of the breeds being crossbreeds for these factors to be detected as significant. The current study excluded visceral MCTs and those tumors closely associated with mucous membranes, and these tumors have been previously reported to have a more aggressive behavior [54, 56, 57].

The current study used a cohort of dogs under primary practice care that had undergone additional prognostic testing. It would therefore be expected that this study would include higher proportions of low to intermediate grade tumors and fewer tumor‐related deaths than a sample selected from a referral institution, due to low and intermediate grade tumors being more common in the general population [3, 4, 5, 6, 7, 58]. However, similar proportions of Patnaik grade I and II, Kiupel low grade tumors were included in this study compared to previous studies [3, 4, 5].

One of the main limitations of the current study was that the histopathology was not reviewed and data was collected from laboratory reports that were returned to the primary care practice. Therefore, different pathologists would have been grading these tumors, leading to a degree of variability between pathologists [59]. Another important limitation of this study was its retrospective nature. Follow‐up data were collected from the contemporaneous clinical records rather than a questionnaire to limit recall bias; however, records were often incomplete, for example, due to dogs moving to another practice or failure to state the reason for euthanasia. It is therefore possible that the number of MCT‐related deaths was underestimated in this study. Initially, statistical models using all‐cause mortality were created; however, many of the dogs included were older, and with the generally low mortality rate in MCTs in dogs, reported between 15% and 30% [3, 4, 5, 8], use of all‐cause mortality could have introduced error by erroneously linking prognostic factors to deaths that were not due to the MCT (type I error). Alternatively, assuming non‐MCT‐related deaths would be distributed randomly with regard to the prognostic panel results, this could have introduced type II error whereby the prognostic variables for MCT‐related mortality were not detected. We could also not accurately extract data on some factors previously reported as prognostic, such as the size of the primary tumor (measured on clinical examination), the speed of its growth, and how long it had been in situ [11, 42, 60], since these features were seldom recorded. Several dogs did undergo adjunctive medical therapy under the care of their primary care vet, and the current study attempted to control for these effects on the results by including adjunctive treatment as a variable in all of the multivariable models. Metastasis was infrequently identified, but this study uses a group of dogs from primary care practices; many dogs (circa 80%–90%) had no staging or very limited staging, and so metastasis could not be included as an outcome measure. The lack of staging and repeat staging in the months or years following surgery might also have resulted in some deaths that were due to MCT metastasis not being diagnosed at the time, leading to an underestimation of MCT‐related death. This could be especially true for dogs with abdominal (either lymph nodes, or viscera) metastasis, as these changes might not be picked up on clinical exam. Finally, the number of dogs that died from tumor‐related causes was low, and this reflects MCTs in general [3, 4, 7, 8] but drawing conclusions from a small number of deaths is difficult.

In conclusion, this study suggests a greater clinical value of *c‐kit* mutation status in exon 11 and AgNOR in the prognostication of all MCTs in dogs, and a lesser importance of Ki67 than previously reported. This study also provides further support for considering a lower mitotic count cut off of ≥ 2 in 10 high‐powered fields for discriminating higher‐risk tumors. However, no single test was consistently shown to independently predict the prognosis of every tumor within a large panel of prognostic tests.

## Disclosure

Authors declare no off‐label use of antimicrobials.

## Ethics Statement

Approved by the ethical committee at CVS UK, approval number 46. Authors declare human ethics approval was not needed.

## Conflicts of Interest

The authors declare no conflicts of interest.

## Supporting information


**Table S1:** full list of breeds included and the genotype group they were included in for statistical analysis.
