# Supplementary material for: Clinical Value of Various Histological Factors in Cutaneous and Subcutaneous Mast Cell Tumors in 197 Dogs
Source: J Vet Intern Med. 2025 Oct 15;39(6):e70244. doi: 10.1111/jvim.70244 (PMC12528809; doi:10.1111/jvim.70244)
Supplement: Supplementary file 1 — Table S1: full list of breeds included and the genotype group they were included in for statistical analysis. [file JVIM-39-e70244-s001.docx]

Supplementary Table 1, full list of breeds included and the genotype group they were included in for statistical analysis

| Breed genotype group | Breed | Number of included dogs |
| --- | --- | --- |
| **Terrier/Brachycephalic** |  | **60** |
|  | Staffordshire Bull Terrier | 22 |
|  | Boxer | 13 |
|  | Boston Terrier | 6 |
|  | French Bulldog | 5 |
|  | Jack Russel Terrier | 3 |
|  | Yorkshire Terrier | 3 |
|  | Mastiff | 2 |
|  | American Bulldog | 1 |
|  | Cairn Terrier | 1 |
|  | Tibetan Terrier | 1 |
|  | Patterdale Terrier | 1 |
|  | Scottish Terrier | 1 |
|  | Border Terrier | 1 |
| **Retriever/Mountain dog** |  | **42** |
|  | Labrador Retriever | 29 |
|  | Golden Retriever | 9 |
|  | Nova Scotia Duck Tolling Retriever | 2 |
|  | Great Dane | 1 |
|  | Rottweiler | 1 |
| **Spaniel/Poodle/Toy** |  | **23** |
|  | Pug | 7 |
|  | English Cocker Spaniel | 5 |
|  | English Springer Spaniel | 5 |
|  | Chihuahua | 3 |
|  | American Cocker Spaniel | 1 |
|  | Beagle | 1 |
|  | Shih-Tzu | 1 |
| **Pastoral including German shepherd dogs, Shar Pei and Husky** |  | **13** |
|  | Miniature Schnauzer | 3 |
|  | Whippet | 2 |
|  | Border Collie | 2 |
|  | Siberian Husky | 2 |
|  | Shetland Sheepdog | 1 |
|  | German Shepherd | 1 |
|  | Shar-Pei | 1 |
|  | Maltese | 1 |
| **Unknown** |  | **20** |
| **Crossbreed** |  | **39** |
